# Expression reflects population structure

**DOI:** 10.1371/journal.pgen.1007841

**Published:** 2018-12-19

**Authors:** Brielin C. Brown, Nicolas L. Bray, Lior Pachter

**Affiliations:** 1 Department of Computer Science, University of California Berkeley, Berkeley, California, United States of America; 2 Institute for Innovative Genomics, University of California Berkeley, Berkeley, California, United States of America; 3 Department of Molecular & Cell Biology, University of California Berkeley, Berkeley, California, United States of America; 4 Division of Biology and Biological Engineering, California Institute of Technology, Padadena, California, United States of America; University of Chicago, UNITED STATES

## Abstract

Population structure in genotype data has been extensively studied, and is revealed by looking at the principal components of the genotype matrix. However, no similar analysis of population structure in gene expression data has been conducted, in part because a naïve principal components analysis of the gene expression matrix does not cluster by population. We identify a linear projection that reveals population structure in gene expression data. Our approach relies on the coupling of the principal components of genotype to the principal components of gene expression via canonical correlation analysis. Our method is able to determine the significance of the variance in the canonical correlation projection explained by each gene. We identify 3,571 significant genes, only 837 of which had been previously reported to have an associated eQTL in the GEUVADIS results. We show that our projections are not primarily driven by differences in allele frequency at known cis-eQTLs and that similar projections can be recovered using only several hundred randomly selected genes and SNPs. Finally, we present preliminary work on the consequences for eQTL analysis. We observe that using our projection co-ordinates as covariates results in the discovery of slightly fewer genes with eQTLs, but that these genes replicate in GTEx matched tissue at a slightly higher rate.

## Author summary

Increasingly complex, high dimensional, multi-modal genomics datasets warrant investigation into analysis techniques that can reveal structure in the data without over-fitting. Here, we show that the coupling of principal component analysis to canonical correlation analysis offers an efficient approach to exploratory analysis of this kind of data. We apply this method to the GEUVADIS dataset of genotype and gene expression values of European and Yoruba individuals, finding as-of-yet unstudied population structure in gene expression abundances. We show that this structure is not driven by known eQTLs, and explore the consequences of our results for eQTL studies involving multiple populations.

## Introduction

Genes mirror geography to the extent that in global populations without admixture, individuals can be localized to within hundreds of kilometers purely on the basis of their genotype [1–3]. Population structure in genotypes is revealed via projection of single nucleotide polymorphism (SNP) data onto the first few principal components of the population-genotype matrix. The principal components space, which is a lower-dimensional distinguished subspace of the high-dimensional data, is computed by a procedure called principal components analysis (PCA). While PCA has been successful in revealing population structure from SNP data, it does not identify such structure in some other genomic data types. For example, in the case of gene expression data, PCA has not revealed obvious population signatures (Supporting Information Fig 1A, [4]). Here we show that although the first two principal components of expression data do not capture population structure, there are other projections that do. One approach to finding such a projection is the coupling of dimension reduction to correlation maximization. This approach, utilizing PCA and canonical correlation analysis (CCA), has been used to effectively analyze the relationship between gene expression and copy number variation [5]. The method is implementable via singular value decomposition and is therefore also efficient. We apply it to finding population structure in expression data, thereby further highlighting the combination of PCA and CCA as a powerful approach to integrative analysis of genomics data. For convenience of notation, we refer to this method as principal component correlation analysis (PCCA).

**Fig 1:**
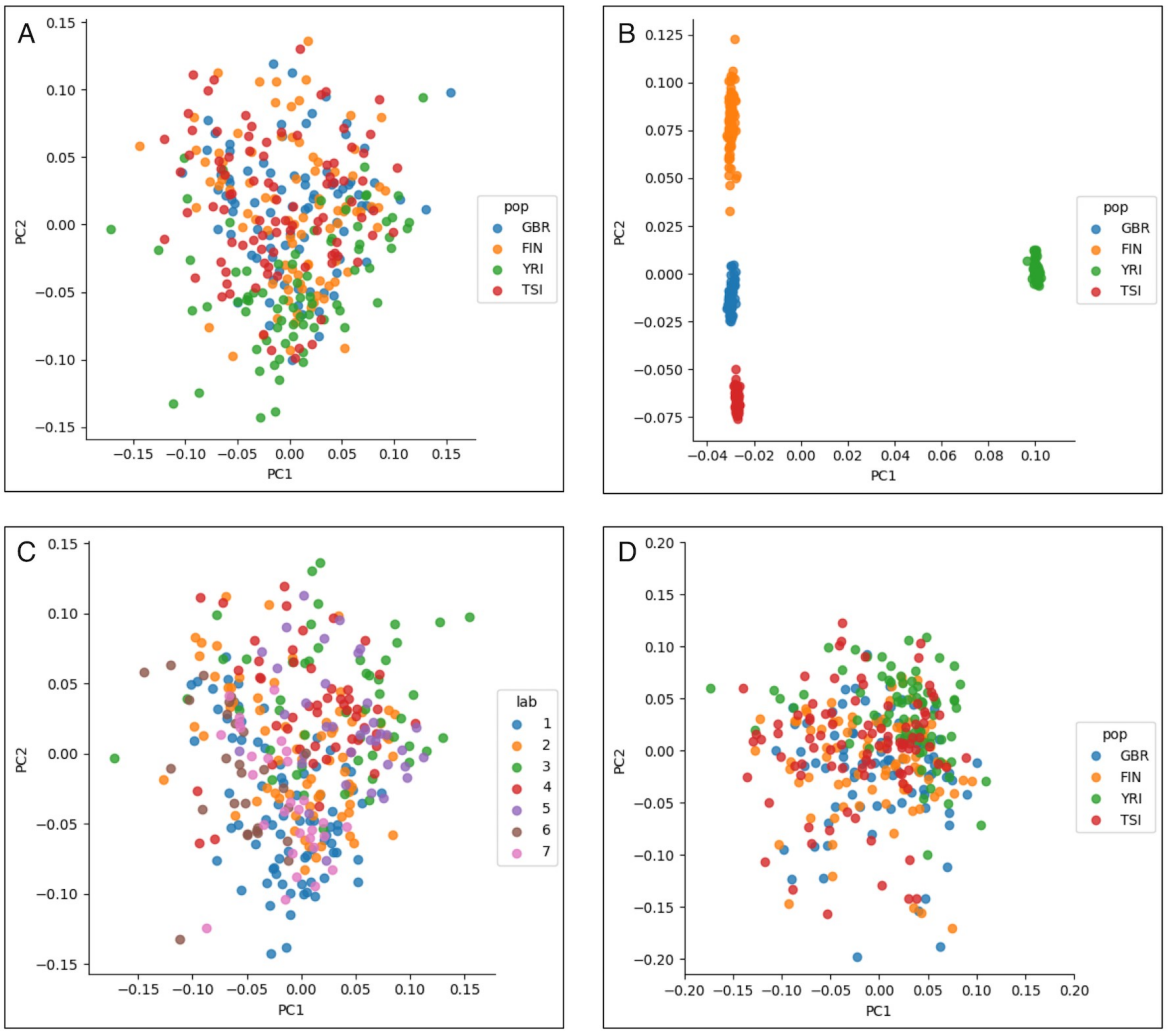
PCA and batch structure within the dataset. (A) PCA of the expression matrix fails to reveal clustering by population, whereas (B) PCA of the genotype matrix reveals clear clustering by population. (C) Coloring of samples by batch reveals that PC1 and PC2 are being partly defined by batch source. (D) After correcting for batch, PCA of the expression matrix still fails to show obvious population structure.

As an optimization procedure, PCA can be viewed as the projection of data onto the lower-dimension subspace that minimizes the average distance of the data to its projection. This is algebraically equivalent to finding the lower-dimensional subspace that maximizes the variance of the projected data [6]. This statistical view of PCA helps to explain why PCA of expression data might not reveal population structure: even if such structure is present in the data, it may not lie on the directions of maximal variance (Fig 1A). CCA is a widely used method for joint analysis of heterogeneous data and provides a linear-algebraic mechanism for identifying shared structure among a pair of datasets. Given a pair of data matrices, CCA finds maximally correlated linear combinations of the columns of each matrix [7]. We show that CCA applied to the PCA projections of expression and genotype data identifies a projection of the expression data that reveals population structure.

To validate our method, we examined population structure in expression data from the Genetic European Variation in Health and Disease (GEUVADIS) project [8], which consists of RNA-seq data obtained from lymphoblastoid cell lines derived from whole-genome sequenced individuals belonging to five distinct populations. From this data, we study 14,070 genes and 6,785,201 SNPs in the Great British (GBR), Finnish (FIN), Tuscan (TSI) (collectively referred to as EUR) and Yoruba (YRI) samples (see Methods). We choose to use the first 30 principal components of expression, the first 5 components of genotype, and the first two canonical correlations (see Methods). The GEUVADIS data has been extensively studied [8–11], yet our analysis reveals structure not previously examined in this well-characterized dataset. In addition to presenting and cross-validating the PCCA projection, we also show that this projection can be constructed from only a small fraction of randomly selected genes and SNPs in the dataset, that it is not primarily driven by allele frequency differences at known cis-eQTLs, and we briefly explore the consequences for multi-population cis-eQTL analyses.

## Results

### Overview of method

A naïve PCA analysis of the GEUVADIS expression data (Fig 1A) shows that unlike genotype data (Fig 1B), there is no clear clustering of individuals by population. This result is consistent with other analyses of expression data, in which population structure is not detected by PCA [4]. To understand the sources of variation that could explain the first and second principal component axes, we labeled the individuals according to the lab where they were sequenced (Fig 1C). This provides some insight into the sources of variation. For example, samples from Lab 3 are distinctly separated from Lab 1. We therefore proceeded to correct for confounding by regressing the gene expression matrix on a matrix of potentially confounding variables and taking the residual (see Methods). After correction for batch, the PCs of the expression matrix still fail to show obvious population structure (Fig 1D). We note that it is also possible to correct for confounding using CCA by exploiting the relationship between CCA with categorical data and linear discriminant analysis [12] (Supplementary Methods, S1 Fig).

Next, we examined whether coupling of expression data to genotype data could identify a projection that reveals population structure. A naïve CCA analysis of genotype and expression again results in a projection that does not reveal population structure, while also suffering from extreme over-fitting since both datasets have many more features than samples (S2 Fig). Instead, we performed PCA followed by CCA on the batch-corrected expression matrix and the genotype matrix. In brief, let *X* be the genotype matrix and *Y* be the expression matrix. Let *U*_*X*,*k*_ be the first k = 5 genotype principal components and U_Y,j_ be the first j = 30 expression principal components. Let $U_{M}\rho V_{M}^{T}=U_{X,k}^{T}U_{Y,k}$ be the singular value decomposition of $M=U_{X,k}^{T}U_{Y,j}$. Then the coordinates of the expression data Y in PCCA space are *C*_*Y*_ = *U*_*Y*,*j*_*V*_*M*,*l*_, where *V*_*M*,*l*_ represents the first l = 2 right singular vectors of M. See Methods and Supplementary Methods for details.

### Population structure in gene expression

**Fig 2:**
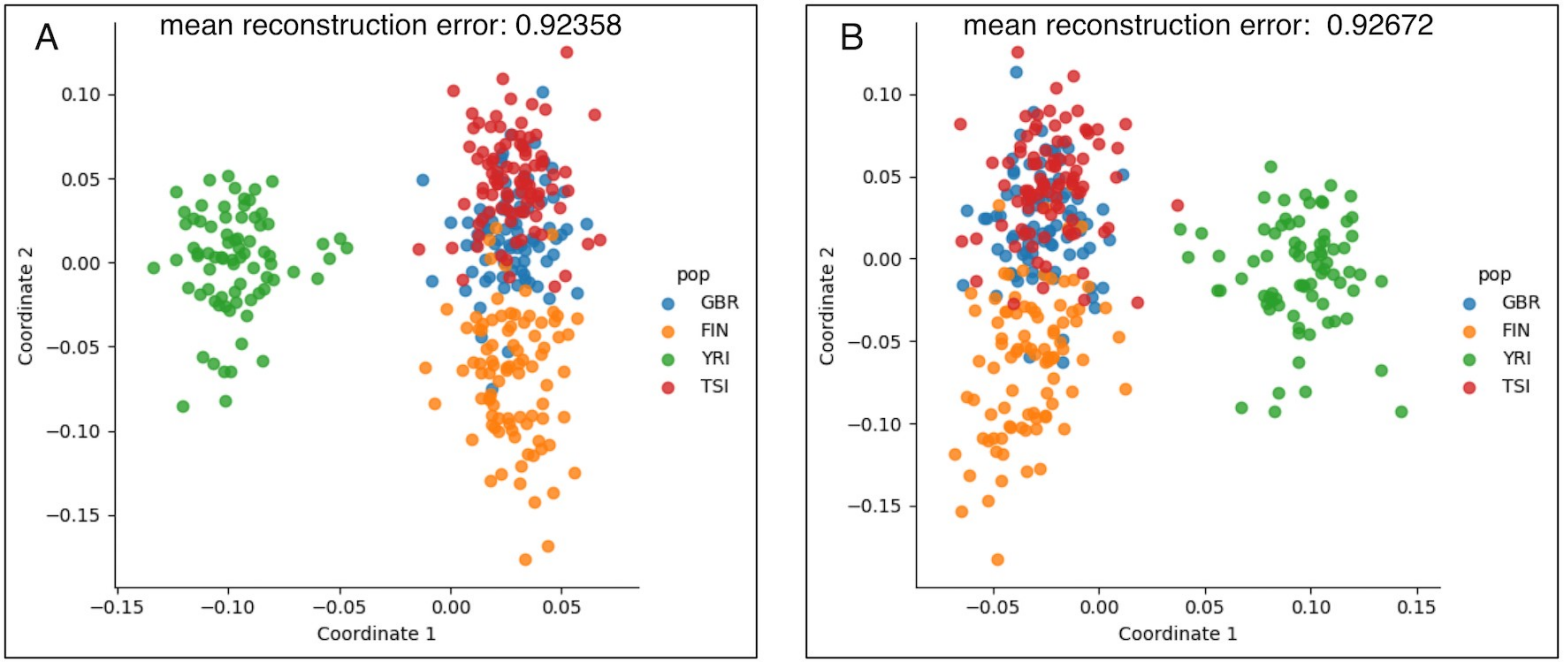
PCA and CCA reveals population structure. (A) A PCCA projection of the batch-corrected expression matrix that shows that expression reflects population structure. While the individuals, labeled according to their population, do not cluster as clearly as with genotype data (Fig 1B), there is clear population structure in the PCCA projection of the batch-corrected expression data. (B) A leave-one-out cross-validation experiment showing that individuals are approximately projected to their populations of origin even when the projection matrix is learned without their expression or genotype data. The mean re-construction errors in (A) the left-in samples and (B) the held-out samples are similar and overlayed on top of the Figure. The first two canonical correlations are 0.963 and 0.766.

The resulting CCA projection of expression data (Fig 2A), reveals distinct population patterns in the data, although not as clearly as the PCA of the genotype data (Fig 1B). The first two canonical correlations are 0.963 and 0.766. To ensure that we did not over-fit, we performed a leave-one-out cross-validation experiment, where we removed each individual from the dataset to confirm that the reconstruction error of the model on the held out point is close to the error in the training set, and that the principal components of the reconstructed gene expression matrix show similar population patterns (Fig 2B). Notably, correction for batch effects, i.e. confounding that is induced by differential sample processing, may not be strictly necessary when applying this method since the batch effects should not be correlated with genotype. In this specific case, two of the seven labs processed 39 of 89 YRI samples, but an application of our method with no correction for lab id gave nearly identical results (S3 Fig).

Since population identity can often be determined using only a handful of SNPs [13], we asked whether the same structure might be visible when using a small number of SNPs, genes, or both. First, we used all genes and randomly sampled SNPs with probability *p* = 0.00001, leaving only 63 total SNPs. With this dataset, we still observe separation of the YRI and FIN populations (S4A Fig). Next, we included all SNPs and randomly sampled genes with probability *p* = 0.01, leaving 142 genes for analysis. In this case we again observe separation of the YRI and FIN populations (S4B Fig). When sampling SNPs with probability *p* = 0.00001 and genes with probability *p* = 0.01 together (57 SNPs and 145 genes), we still observe separation of the YRI population, but not the FIN population (S4C Fig). Remarkably, by increasing the sampling rate for SNPs and genes to *p* = 0.00002 and *p* = 0.02, respectively (111 SNPs, 283 genes), we again recover the separation of YRI and FIN populations, demonstrating that population structure can be identified using only a small fraction of SNPs and genes (S4D Fig).

### Identifying genes that contribute to population structure

**Fig 3:**
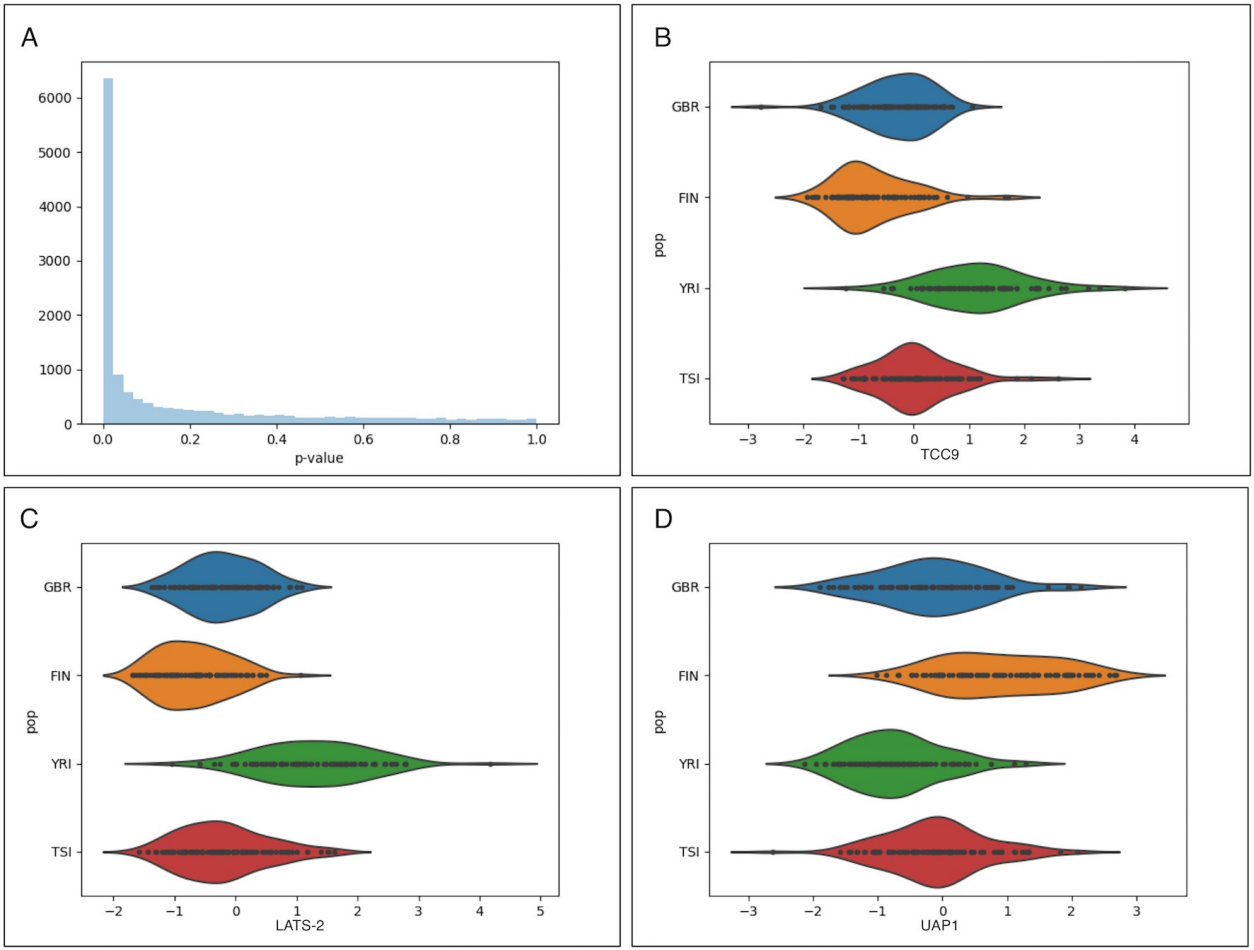
Visualizing important genes. (A) The *p*-value distribution for tests that the variance of each gene in the projection is greater than the null shows a large number of genes with significant scores in the PCCA projection. The expression distributions by population for the three genes with highest *z*-scores are shown in (B) the LATS-2 gene, (C) the EIF4EBP2 gene, (D) the STX7 gene.

The CCA projection is indexed by linear combinations of genes, which can be understood to discriminate individuals based on expression signatures. That is, genes with high variance in the CCA expression projection (see Supplementary Methods) have expression distributions that segregate based on patterns in the genotype PCs, which we interpret to represent population structure [1–3]. After correction for correlated multiple testing using the Benjamini–Hochberg–Yekutieli procedure [14], we identified 3,571 genes with significant scores at FDR 5%, indicating that population structure within gene expression data is pervasive. The three genes with largest *z*-score in this analysis were TCC9, LATS-2 and UAP1 (Fig 3). The first two genes display increased expression in the YRI population, whereas the third displays increased expression In the FIN population.

**Fig 4:**
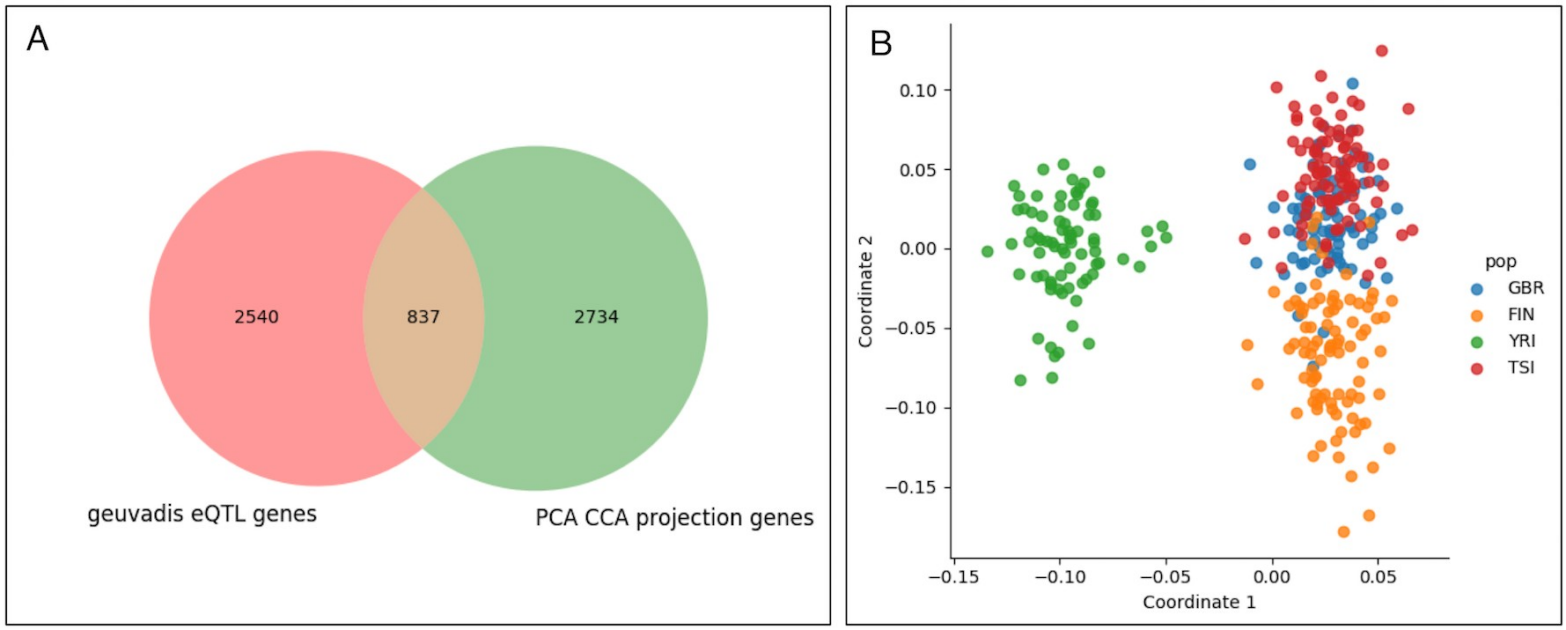
Comparison to GEUVADIS results. (A) A Venn diagram showing the overlap between GEUVADIS eQTL genes and genes that significantly influence the PCCA projection, showing that 837 of the PCCA genes were determined to be eGenes in the GEUVADIS analysis. (B) Removing the population mean effect of the lead eQTL SNP for all GEUVADIS eGenes has no perceptible effect on the PCCA projection. In this case, the first two canonical correlations are 0.966 and 0.803.

After identifying genes that significantly influence the PCCA projection, we sought to contextualize our result within the original GEUVADIS eQTL analysis. The GEUVADIS analysis identifies 3,377 genes with an eQTL (eGenes) in either the EUR or YRI populations. Of these, 2,539 are among the 14,070 genes used in our analysis. We found that 837 of these genes were determined to be significant in our analysis, and therefore that 2,734 of our significant genes were not reported as eGenes in the original GEUVADIS analysis (Fig 4A).

To further evaluate the effects of known eQTL variants on our analysis, we removed the population-level expected gene expression level from each gene with a GEUVADIS eQTL (see Methods). We then re-normalized the expression values and re-calculated the PCCA projection with genotype (Fig 4B). We observe no perceptible difference between the main projection and the projection after removing the population-level expected gene expression level, and little change to the canonical correlation values (0.966 and 0.803). This indicates that genes with known eQTLs in the GEUVADIS analysis are not the primary drivers of the PCCA structure.

### Implications for eQTL analysis

Finally, we sought to understand the implications of the PCCA projections for eQTL studies involving multiple populations. We conducted two joint cis-eQTL analyses of the four GEUVADIS populations examined in this study. In both cases, we used the common strategy of correcting for the top 10 PEER factors [8,15] and regressing each gene level on every SNP with MAF > 5% within 1 MB of the transcription start site (TSS), independently. In the first study, we included the first 5 principal components of the genotype matrix as covariates in the analysis (the PC strategy), and in the second we used the first 5 components of both the gene and genotype PCCA projection as covariates (the PCCA strategy).

**Fig 5:**
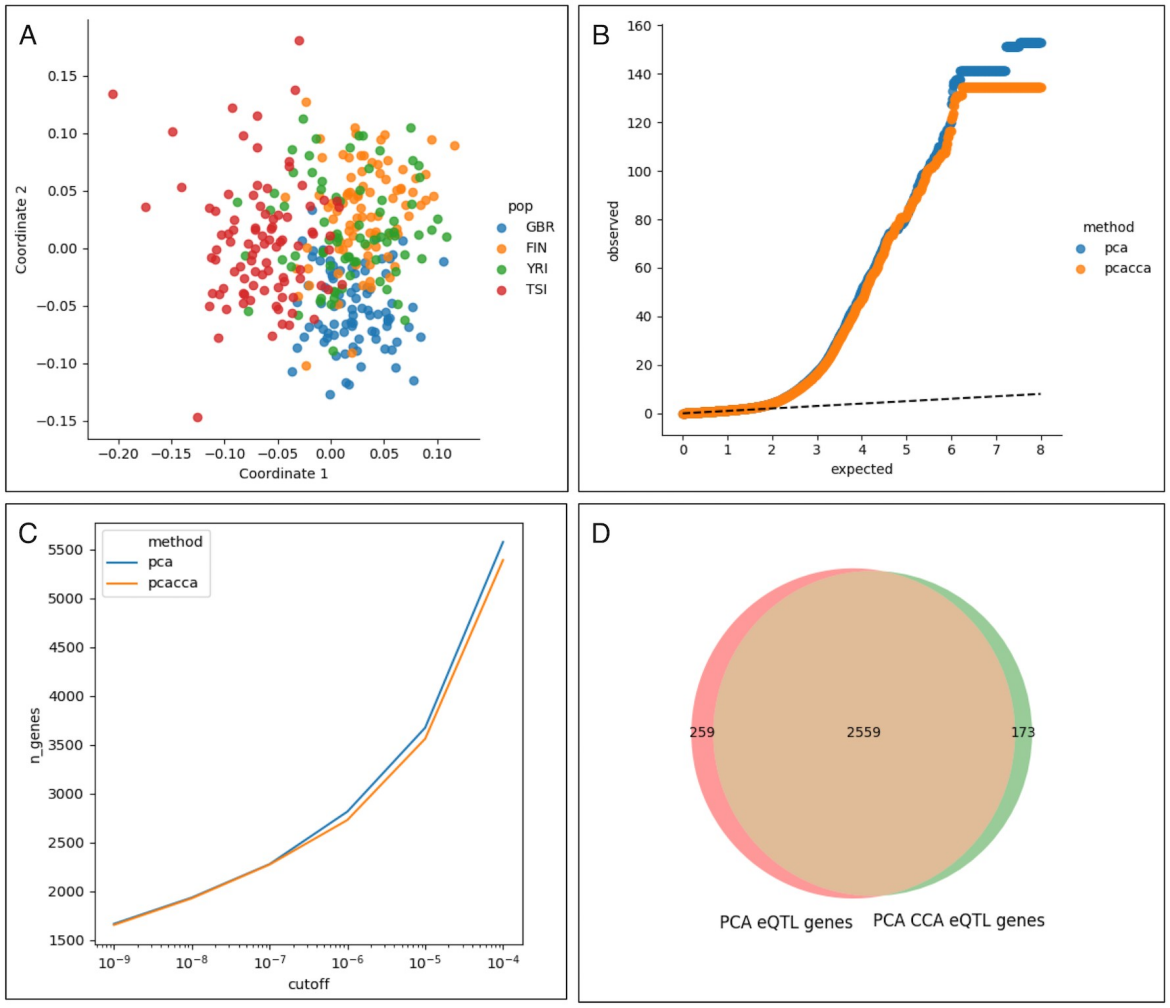
Consequences for eQTL analysis. A comparison of a standard eQTL pipeline when using either the first PCs of genotype or the first gene and genotype PCCA coordinates as covariates in the regression. (A) When using PEER to correct for batch effects, the separation of the YRI population is removed while the structure within the EUR populations remains. (B) The number of genes with an eQTL as a function of the significance cutoff for both methods, showing that the PCCA approach discovers slightly fewer genes at all levels. (C) A Q-Q plot of–log10(p) for the eQTL results using either method against a uniform distribution, showing reduced inflation at the high end. (D) Overlap of the genes discovered by the two methods at a nominal significance level of α = 1e-6. Though the overlap is large, the genes discovered using PCCA co-ordinates as covariates are not a strict subset of the genes discovered using PCs of genotype as covariates.

Interestingly, we found that using PEER rather than regression for batch correction also removed the separation between the YRI and EUR individuals, while leaving the structure within the EUR populations in tact (Fig 5A). In addition, a Q-Q plot of the p-values resulting from the eQTL analysis shows reduced inflation at the top end of the distribution when using the PCCA strategy as opposed to the PC strategy (Fig 5B). Next we compared the number of eGenes discovered using both methods of correction as a function of the nominal significance cutoff used. For all significance cutoffs analyzed, we found slightly fewer eGenes using PCCA coordinates as covariates (Fig 5C). For example, at a nominal significance value of α = 1e-6, we found 2,818 eGenes using the PC strategy and 2,732 eGenes using the PCCA strategy.

The eGenes discovered using the PCCA strategy are not a strict subset of the eGenes discovered using the PC strategy (Fig 5D). At the same nominal significance of α = 1e-6, the PC strategy discovers 259 eGenes not discovered using the PCCA strategy, while the PCCA strategy discovers 173 eGenes not discovered using the PC strategy. To compare the accuracies of the two methods, we used GTEx EBV-transformed lymphocyte eGenes as a replication dataset (Table 1). Of the 2,818 eGenes discovered using the PC strategy, 1,407 were reported as significant at FDR 10% in the GTEx dataset (49.92%), while 1,394 of the 2,732 eGenes discovered using the PCCA strategy were reported as significant at FDR 10% (51.02%). We compared replication Q-value cutoffs from 0.05 to 0.50, and found that at all cutoffs used, eGenes discovered using the PCCA strategy replicated at a slightly higher rate (Table 1).

**Table 1:** Replication rate of genes discovered using the PC strategy and the PCCA strategy in GTEx EBV-transformed Lymphocytes as a function of the replication Q-value cutoff.

Q-value	0.05	0.10	0.20	0.30	0.40	0.50
PCA	42.79%	49.92%	62.85%	75.05%	86.65%	94.42%
PCCA	43.70%	51.02%	64.34%	76.35%	87.18%	94.72%

## Discussion

A key feature of the PCCA approach is interpretability in the form of genes which significantly influence the projections, highlighting the possibility of directly relating population expression differences to disease as in [16]. One interesting example is the gene PSPH (p<1e-7), which was examined in [17] and was found to be the gene with the highest degree of differential allelic expression. This gene is reported as an eGene in the original GEUVADIS EUR analysis but, importantly, not the GEUVADIS YRI analysis. The reported eQTL in that analysis is rs34458430. The SNP rs6700, which has also been reported as an eQTL for that gene, is an ancestry informative marker [18]. In [19] authors show that PSPH plays an important role in breast tumor development, and in [20], the authors note that elevated PSPH levels in breast tumors give poor prognosis, and that PSPH is elevated in tumor samples from African American women. We wondered whether any SNP in the region near rs6700 (chr 7:55,773,495) was associated with breast cancer, and found that rs12718945 (chr7:55,125,270) was reported as such in [21]. While rs12718945 is not in LD with rs6700 or rs34458430, it does have different allele frequencies in the YRI and EUR GEUVADIS populations. Specifically, in YRI the effect allele T has a frequency of 68%, while in EUR this allele has a frequency of 48%. However, rs12718945 is nearly 1MB away from the PSPH transcription start site (chr7:56.078,056) and therefore is excluded from most cis-eQTL analyses.

While we view the identification of such genes as important, we caution that African-Americans also experience substantial structural inequality in healthcare, which confounds this analysis [22]. We also note that while genes such as PSPH must also have substantial genetic/epigenetic regulation that is linked to population differences, the projection-associated genes identified by our method does not produce that information. Indeed, its power to detect genes associated with population structure comes by virtue of requiring only one test per gene and is agnostic to the source of regulation. While a complete analysis of population-associated expression differences is beyond the scope of this paper, this example suggests that our method should be a powerful approach for directly identifying genes whose expression associates with population.

With the observation that the directions of maximal variance in the gene expression data do not represent population structure or even technical variation, we wondered what they did represent. We calculated the variance in the first two PCs of every gene and searched for the top 100 using the Gene Ontology PANTHER Overrepresentation test for biological process in homo sapiens, database release 2018-10-08 [23,24]. Using Fishers exact test with a Bonferroni correction, we found significant results for the top-level categories “regulation of gene expression” (p<0.0261), “regulation of RNA metabolic process” (p<0.0392), and “regulation of cellular macromolecule biosynthetic process” (p<0.032). This indicates that the directions of maximal variance capture basic components of gene and metabolic process regulation (see S5 Fig for full GO output).

We have shown that many genes (3,571) contribute to population structure, and that the majority of these (2,734) were not reported as eGenes in the original GEUVADIS analysis. Moreover, we have demonstrated that removing the population-level expected expression due to these genes yields nearly identical visualizations. While some may view this as unexpected, there could be a number of reasons for this. First, we show that only a few hundred genes can be used to produce visualizations that separate out the FIN and YRI populations and therefore removing some signal from only 6% of the genes is unlikely to effect this. Second, most cis-eQTL analyses separate African populations and attempt to control for population structure as much as possible, whereas we explicitly look for genes that separate by population. Third, it has been shown that the genetic correlation of eQTL effect sizes between YRI and EUR populations in GEUVADIS is low [25], and therefore applying the effect sizes learned from the EUR population to the YRI population and vice versa may be problematic.

It is possible that with full knowledge of cis-genetic effects on gene expression, population-level differences in expression could be entirely explained by differences in allele frequency at these variants. It is also possible that genetic effects on gene expression are so pervasive, and gene networks so interconnected, that nearly every gene is affected by genetic variation in trans from thousands of variants. This is consistent with the recently described omnigenic model [26], and many studies showing that the majority of heritability of gene expression is explained in trans [27–31]. Under this model, population-level expression differences could be explained by consistency in effect from many eQTLs acting in trans. We view exploration of the differential contributions of cis and trans eQTL effects on population structure in gene expression as an intriguing area for future research.

However, methods that improve power to detect cis-eQTLs while handling data from multiple populations remain important. We have explored the consequences of our result for eQTL analyses in multiple populations by using the coefficients from our model as covariates in the analysis. While we discover fewer genes with this method, the genes we do discover replicate at a slightly higher rate in a matched GTEx tissue. We caution that the observed difference in replication rate is very small and that these results are preliminary. Further investigation including simulations and testing in additional, larger eQTL cohorts will be required before we can definitively say that this is a superior approach to eQTL analysis.

The identification of population structure in expression data suggests that it should be interesting to extend population genetic methods such as [32] to population transcriptomics. The example of joint analysis of expression and genotype data can be extended to include other data types via an extension of CCA to more than two matrices [12,33–35], and the coupling of PCA to CCA could also be extended to a hierarchical factor analysis method. Importantly, the coupling of PCA and CCA is not the only projection that reveals population structure. For example, connecting the principal components using linear regression gives similar visualizations (Supplementary Methods, S6 Fig). The choice of model should reflect the variance structure of the data, which here we have deliberately remained agnostic to. Moreover, there are other variants of CCA that can be used to analyze genomic data, such as sparse and regularized CCA [5,34]. Ultimately, it is important to identify the optimal model for inference.

While we believe the extensions described above will be interesting to pursue, our analysis and that in [5] show that PCCA is a useful and rapid approach to exploratory analysis of heterogeneous data. As the generation of large-scale, high-dimensional, multi-modal genomics datasets becomes more commonplace [35–37], we expect the combination of PCA and CCA to become as common as PCA is today.

## Methods

We obtained genotype data of the Phase 1 1000 genomes individuals in PLINK format [38] from cog-genomics [See Data and Software Availability]. GEUVADIS project RNA-seq reads were downloaded from the European Nucleotide Archive (accession number ENA: ERP001942). In the analyses performed we omitted the CEU population because it has been previously found to display biased expression patterns due to the age of the cell line [10]. Importantly, this bias affects every CEU sample and therefore cannot be corrected for traditional methods of handling confounding.

There are 343 individuals with genotype data from 1000 genomes phase 1 and corresponding RNA-seq data from GEUVADIS in the FIN, GBR, TSI and YRI populations. We quantified the transcript abundances of these individuals using kallisto [39] with the GENCODE v27 protein coding transcript sequences annotation. The GENCODE v27 annotation contains 95,659 transcripts. We omitted all transcripts with mean transcripts per million (TPM) less than 0.1 across the quantified samples, leaving 58,012 transcripts. We then used the GENCODE v27 annotation to obtain gene level quantifications by summing transcript quantifications in TPM units. Finally, we removed genes in the MHC region and on non-autosomal chromosomes. This left 14,070 genes for analysis. The Phase 1 1000 genomes genotypes contain 39,728,178 variants. We filtered indels, variants with minor allele frequency (MAF) less than 5%, and non-biallelic SNPs leaving 6,785,201 SNPs for analysis. Finally, we quantile-normalized the expression matrix, and centered and scaled each gene quantification vector to have mean 0 and variance 1. In the following analyses, we chose to keep 30 principal components of expression and 5 principal components of genotype, while analyzing the first two canonical components (Fig 2). We chose these numbers by inspecting a plot of the percentage of variance explained as the number of components is increased, also known as the elbow method (S7 Fig). However we note that our results are stable under different choices of numbers of components (S8 Fig, S1 Table). In that analysis, we choose to use a smaller number of PCs of genotype than expression due to the observation that the genotype data has a smaller number of large eigenvalue components than the expression data (S7 Fig). Intuitively, one can imagine the population structure in the genotype data dominates the first few PCs, while it is spread out more among the top PCs of the expression data.

To remove batch effects from the expression matrix, we one-hot encoded the lab identification vector, and then added a column for sample gender [40], resulting in a 343 *x* 7 matrix of potentially confounding variables. We then regressed each gene expression vector on the confounding matrix and used the residual expression vector for all further analysis. Next, we computed principal components of the genotype matrix using PLINK and principal components of the corrected expression matrix using the eigendecomposition of the Gram matrix (See Fig 1 for visualizations). Finally, we computed the canonical variables between the top principal components of the genotype and corrected expression matrices (see the Supplementary Methods for details on the linear algebra).

To verify that we did not over-fit in estimating coefficients using CCA, we performed leave-one-out cross validation. We removed each of the 343 individuals one-by-one from the dataset, re-calculated the principal components of the genotype and expression matrices, and re-estimated the canonical variables and bases. We then projected each held out individual into the resulting CCA gene expression subspace. After this process, for each individual, we plotted the first two principal components of the re-constructed expression matrix to verify the individual clusters by population (Fig 2B, see also the Supplementary Methods for details of the how the projection was performed). Furthermore, we calculated the in-sample and out-of-sample reconstruction error as the squared Frobenius norm of the original and reconstructed data points, and verified that it was similar for both left-in and held-out samples.

We asked which genes had significant variance in the CCA gene expression projection. We computed the variance of each gene in the projection, and calculated significance via a permutation test with 10 million permutations. In each iteration, we shuffled the genotype principal components and recomputed the variance explained. The *p*-value derived from this test is the number of times the permuted score is greater than the observed score, divided by the number of permutations (see the Supplementary Methods for details of how the variance is computed). We further estimated a *z*-score for each gene as the difference between the estimated and mean permutation variance divided by the variance of the permuted variance.

To remove the effects of known GEUVADIS eQTLs, we downloaded the YRI and EUR summary statistics [8]. The authors provide the correlation of genotype and expression for the top SNP identified at each gene determined to be significant at FDR 5% (*r*_*g*_). For any e-gene reported in both the YRI and EUR datasets, we chose the larger *r*_*g*_ value. From this correlation, we calculate the effect size as $\beta_{g}=\frac{r_{g}}{\sqrt{2f_{a}(1-f_{a})}}$ where *f*_*a*_ is the allele frequency of the associated variant in all populations considered. From this we computed the mean expected population expression level for gene *g* as *Y*_*G*,*k*_ = 2*β*_*g*_*f*_*k*_ where *f*_*k*_ is the frequency of the associated variant in population *k*. We subtracted this value from the empirical expression level for each e-gene for every individual in population *k* and recalculated the projection.

To conduct our cis-eQTL analysis, we corrected for batch effects using the first 10 PEER [15] factors and used PLINK [38] to do the regression analysis. We used the plink “—linear” association method for every SNP with allele frequency above 5% in the combined EUR+YRI dataset within one megabase of the TSS of each gene. For the PC strategy we used the first 5 principal components of genotype as covariates and for the PCCA strategy we used the first 5 genotype and first 5 expression PCCA components as covariates.

## Data and software availability

The software used to produce the analyses is on GitHub. We provide a package of tools for computing the projections and estimating gene significance, as well as a Snakemake file [41] that can be used to completely reproduce the analysis, from data acquisition to figure generation.

- Analysis software: https://github.com/pachterlab/PCCA/
- Gencode v27 transcripts: ftp://ftp.sanger.ac.uk/pub/gencode/Gencode_human/release_27/gencode.v27.pc_transcripts.fa.gz
- Gencode v27 GTF: ftp://ftp.sanger.ac.uk/pub/gencode/Gencode_human/release_27/gencode.v27.annotation.gtf.gz
- GEUVADIS RNA-seq reads: ftp://ftp.sra.ebi.ac.uk/vol1/ERA169/ERA169774/fastq
- 1000 genomes genotypes: https://www.dropbox.com/s/k9ptc4kep9hmvz5/1kg_phase1_all.tar.gz

## Supporting information

S1 Table Different choices of numbers of components give correlated scores. Cross-correlation matrix of the Z-scores obtained for each gene across the different choices of components presented in S8 Fig.
(PDF)

S1 Fig LDA is related to CCA and can be used for correction. (A) CCA between PCs of expression and a confounding matrix is related to LDA, and projection into the learned space reveals strong clustering by batch within the data. (B) Projecting orthogonally to this space leaves samples scrambled by batch in the first PCs. (C) Using this correction instead of regression gives similar results for CCA between PCs of expression and genotype. (D) As in the main text, structure is maintained during a cross-validation experiment.
(PNG)

S2 Fig Using standard CCA with all genes and genotypes results in no population structure and high over-fitting. (A) The results of running standard CCA using all genes and genotypes and (B) the CV-projection in a leave-one-out experiment. In this case, no population structure is identified, and the resulting first two correlation coefficients are both 1.0. Since there are many more columns than samples in both datasets, there are many A,B such that Corr(XA,YB) = 1.0. This is an example of extreme over-fitting, with a train error of 0.992 and a test error of 1.295.
(PNG)

S3 Fig Running PCCA without including batch as a covariate gives nearly identical results. (A) The results of running PCCA without including batch as a covariate. (B) The CV-projection in a leave-one-out experiment on this data. The results are nearly identical to the results when including batch. In this case the first two canonical correlations are 0.964 0.793, respectively.
(PNG)

S4 Fig Subsampling SNPs and genes shows that similar structure can be obtained with a small fraction of genes. The results of running this procedure when subsampling without replacement either (A) SNPs, (B) genes or (C, D) both. In (A), we sample each SNP with probability p = 0.00001 for a total of 63 SNPs, while keeping all genes, and still observe separation of both the YRI and FIN popultions. In (B), we sample each gene with probability p = 0.01 for a total of 142 genes and again observe similar structure. In (C) we sample each SNP with probability p = 0.00001 and each gene with probability p = 0.01 (57 SNPs, 145 genes) and still observe separation of the YRI, but not the FIN population. In (D), we increase this to p = 0.00002 and p = 0.02, respectively (111 SNPs, 283 genes) and again observe separation of both YRI and FIN populations.
(PNG)

S5 Fig Results from a GO enrichment analysis of the genes with the most variance in the projection onto the first two principal components. (PNG)

S6 Fig Using regression rather than CCA to relate the principal components of the two data matrices also yields a projection that reveals population structure within the expression data. (PNG)

S7 Fig Percentage of variance explained as a function of the number of PCs used in expression and SNP data. Percentage of variance in the data explained as a function of the number of principal components for (A) gene expression and (B) genotype. The linear region occurs much earlier in the genotype data, implying that fewer components should be used in this analysis.
(PNG)

S8 Fig Choosing different numbers of PCA components provides similar visualizations. (A) 13 expression and 5 genotype components. (B) 42 gene expression and 15 genotype components. (C) 100 expression and 2 genotype components.
(PNG)
