# Supplementary material for: Expression reflects population structure
Source: PLoS Genet. 2018 Dec 19;14(12):e1007841. doi: 10.1371/journal.pgen.1007841 (PMC6317812; doi:10.1371/journal.pgen.1007841)
Supplement: S1 Table — Cross-correlation matrix of the Z-scores obtained for each gene across the different choices of components presented in S8 Fig. (PDF) [file pgen.1007841.s001.pdf]

Supplementary Methods
Expression reflects population structure

Brielin C Brown, Nicolas Bray and Lior Pachter

October 29, 2018

1 Principal Component Analysis

Let X be an $N \times D$ matrix of rank $r \leq N < D$. The singular value decomposition of X is a factorization of the form $X = U\Lambda V^\top$, where U and V are $N \times r$ and $D \times r$ unitary matrices, respectively, and $\Lambda = diag(\lambda_1, \ldots, \lambda_r)$ is a diagonal matrix containing the singular values of X. The columns of U and V are called the left and right singular vectors, respectively [1]. While keeping the full spectrum of singular values gives an exact decomposition of X, one can instead keep only the top $l < r$ singular values, using U_l and V_l to denote the first l columns of U and V, respectively, and Λ_l to denote the first l singular values. This gives an approximation to X which we call $\tilde{X} = U_l \Lambda_l V_l^\top \approx X$.

Let $\Sigma = \frac{X^\top X}{N-1}$ be the covariance of X. We use $A \cdot_\Sigma B = A\Sigma^+ B$ to indicate matrix multiplication with respect to the Mahalanobis metric with covariance Σ. When X is column-mean subtacted, the principal components of X are the l-dimensional coordinates defined by the rows of U_l. These coordinates have basis $F = \frac{V_l \Lambda_l}{\sqrt{N-1}}$ which are orthogonal with respect to the Mahalanobis metric on X. We note that this definition of principal component analysis (PCA) is slightly different than the classical definition. Specifically, the classical formulation takes $U_l \Lambda_l$ as its coordinates and V_l as the orthonormal Euclidean basis [2]. However in genetics the basis scaling we use here is more common. Specifically, if X is a genotype matrix this is equivalent to defining the principal components as the eigenvectors of the realized relationship matrix as in [3, 4].

Then projection of X into the space spanned by F and its complement are given by

$$\begin{aligned}
\tilde{X} &= X \cdot_\Sigma FF^\top \\
&= X \left(\frac{X^\top X}{N-1} \right)^+ FF^\top \\
&= U\Lambda V^\top V \Lambda^{-2} V^\top V_l \Lambda_l^2 V_l^\top \\
&= U_l \Lambda_l V_l^\top. \\
\tilde{X}^\perp &= X - \tilde{X}.
\end{aligned}$$

Next, we extend these concepts to canonical correlation analysis before combining them to define explicitly the method we use.

2 Canonical Correlation Analysis

Given two data matrices X and Y with the same number of rows representing distinct but related data, canonical correlation analysis (CCA) finds maximally correlated linear combinations of the columns of X and Y. CCA identifies matrices A and B such that the sequence of correlations $\rho_{XY,i} = \text{corr}(XA^{(i)}, YB^{(i)})$, where $A^{(i)}$ is the ith column of matrix A, is successively maximized so that the correlation matrix $\rho_{XY} = \text{corr}(XA, YB)$ is diagonal [2].

Specifically, let X and Y be $N \times D_X$ and $N \times D_Y$ data matrices of rank r_X and r_Y respectively. As in the previous section, we denote the singular value decompositions of X and Y by $X = U_X \Lambda_X V_X^\top$ and $Y = U_Y \Lambda_Y V_Y^\top$. The canonical correlations of X and Y are given by the singular values of the matrix $M = U_X^\top U_Y = U_M \rho_{XY} V_M^\top$. The canonical bases are given by $F_X = \frac{V_X \Lambda_X U_M}{\sqrt{N-1}}$ and $F_Y = \frac{V_Y \Lambda_Y V_M}{\sqrt{N-1}}$, again orthonormal with respect to the Mahalanobis metric on Σ_X and Σ_Y respectively [5]. As with PCA, one can instead keep only the top k singular vectors, which we denote by $U_{M,k}$ and $V_{M,k}$ to indicate the first k columns of U_M and V_M respectively. In this case, the bases are $F_{X,k} = \frac{V_X \Lambda_X U_{M,k}}{\sqrt{N-1}}$ and $F_{Y,k} = \frac{V_Y \Lambda_Y V_{M,k}}{\sqrt{N-1}}$.

The coordinates of the data matrices in k-dimensional CCA space, which we refer to as the canonical variables, are given by

$$
\begin{aligned}
C_{X,k} &= X \cdot_{\Sigma_X} F_{X,k} = U_X U_{M,k}, \\
C_{Y,k} &= Y \cdot_{\Sigma_Y} F_{Y,k} = U_Y V_{M,k}.
\end{aligned}
$$

Similarly, the projections of the data matrices onto the canonical bases are given by

$$
\begin{aligned}
\tilde{X}_{C,k} &= X \cdot_{\Sigma_X} F_{X,k} F_{X,k}^\top = U_X U_{M,k} U_{M,k}^\top \Lambda_X V_X^\top, \\
\tilde{Y}_{C,k} &= Y \cdot_{\Sigma_Y} F_{Y,k} F_{Y,k}^\top = U_Y V_{M,k} V_{M,k}^\top \Lambda_Y V_Y^\top.
\end{aligned}
$$

As with PCA the complement is given by

$$
\begin{aligned}
\tilde{X}_{C,k}^\perp &= X - \tilde{X}_{C,k} = U_X (\mathbb{I} - U_{M,k} U_{M,k}^\top) \Lambda_X V_X^\top, \\
\tilde{Y}_{C,k}^\perp &= Y - \tilde{Y}_{C,k} = U_Y (\mathbb{I} - V_{M,k} V_{M,k}^\top) \Lambda_Y V_Y^\top.
\end{aligned}
$$

From this, it is straightforward to interpret linear discriminant analysis (LDA) as a special case of CCA [6]. Let Y be a matrix of data observations and let L be a length N categorical data vector with K categories. Consider the $N \times K - 1$ indicator matrix X with the ith column the indicator vector $\mathcal{I}[L = i]$, sometimes called the one-hot encoding of the data. Then LDA between X and Y is equivalent to learning the CCA projection $Y_{C,k}$, and one can project out effects correlated with the categories in L by computing $Y_{C,k}^\perp$. Indeed, we can replace the regression correction for batch and gender in the main text with this approach and obtain similar visualizations. See Supplementary Figure 1.

We combine PCA with CCA as in [7] to arrive at our final projection. Specifically, we work with the first l_X PCA components of X and the first l_Y PCA components of Y. In this case, $M = U_{X,l_X}^\top U_{Y,l_Y}$ such that the coordinates in k-dimensional CCA space are

$$
\begin{aligned}
C'_X &= U_{X,l_X} U_{M,k}, \\
C'_Y &= U_{Y,l_Y} V_{M,k}.
\end{aligned}
$$

with bases

$$
\begin{aligned}
F'_X &= \frac{V_{X,l_X} \Lambda_{X,l_X} U_{M,k}}{\sqrt{N-1}}, \\
F'_Y &= \frac{V_{Y,l_Y} \Lambda_{Y,l_Y} V_{M,k}}{\sqrt{N-1}}.
\end{aligned}
$$

As before, the projection of the data matrices onto the canonical bases and their complements are given by

$$
\begin{aligned}
\tilde{X}_{C'} &= X \cdot_{\Sigma_X} F'_X F'^\top_X = U_{X,l_X} U_{M,k} U_{M,k}^\top \Lambda_{X,l_X} V_{X,l_X}^\top \\
\tilde{Y}_{C'} &= X \cdot_{\Sigma_Y} F'_Y F'^\top_Y = U_{Y,l_Y} V_{M,k} V_{M,k}^\top \Lambda_{Y,l_Y} V_{Y,l_Y}^\top \\
\tilde{X}_{C'}^\perp &= X - \tilde{X}_{C'} \\
\tilde{Y}_{C'}^\perp &= Y - \tilde{Y}_{C'}
\end{aligned}
$$

3 Leave-One-Out Cross-Validation

Let the genotype matrix of the sample be X and the expression matrix of the sample be Y. For individual i, the full matrices X and Y are reduced by removing row i to create X_{-i} and Y_{-i}. We then learn the canonical bases F'_X, F'_Y of the data matrices using the combination of PCA and CCA described above. Next we project the held out individual gene vector y^i into this space to get $y^i_C = y^i \cdot_\Sigma F'_{Y,-i} F'^{\top}_{Y,-i}$. After repeating this for all individuals, we form the data matrix Y'_C where each row i is the projection of individual i's gene expression vector into the CCA-gene space learned without using i. A plot of the first two principal components of this data matrix shows that the population structure learned by this method is valid with respect to held-out samples (Figure 2, Supplementary Figure 3).

4 Determining the Significance of a Gene for the CCA Projection

Let $F'_{Y,(j)}$ be the j'th row in the basis matrix F'_Y. The variance v_j for gene j is given by $v_j = ||F_{Y,(j)}||_2^2$. Therefore, to determine whether the variance of a gene is non-null, we can perform a permutation test. Specifically, in each permutation p we shuffle the genotype principal components and compute the permuted variance for each gene v_j^p. The p-value for the test that the variance is greater than the null score is then the number of times the permuted variance is greater than the true variance. That is, for N_p permutations, the p-value is $p_j = \sum_p \mathbb{1}[v_j^p > v_j]/N_p$.

5 A regression based approach

While performing CCA between the projected data matrices is one approach to visualizing the common structure underlying the principal components of two data matrices, there are possibly many projections that yield such a result. One involves assuming that the principal components of one matrix have a linear relationship to the principal components of the other with additive noise. That is if, $U_Y = U_X\beta + \epsilon$, the least squares solution is

$$\hat{\beta} \quad = \quad U_X^\top U_Y.$$

This time, the coordinates of the data matrix in the projection are

$$\begin{aligned} C_{Y,k} &= U_X\hat{\beta} = U_X U_X^\top U_Y, \\ C_{X_k} &= U_X. \end{aligned}$$

Compared to the CCA approach, the regression approach presumes a different noise model for the data. Rather than modeling the principal components of both gene expression and genotype as linearly related to an underlying hidden factor with additive noise, the regression approach implictly models the genotype principal components as the underlying factor that influences gene expression principal components with additive noise. Ultimately, the choice of model should reflect the "noise" structure emerging from the underlying biology and the nature of the measurements. One notable drawback of the regression based approach is that it uses the location of the genotype in genotype-PCA space to approximate the location of the expression vector in expression-PCA space, as opposed to the CCA projection which only needs to know the global relationship between the points in expression-PCA and genotype-PCA space. A consequence of this is that any held-out individual needs to have their genotype projected into genotype-PCA space, which is computationally infeasible when working with millions of SNPs. Therefore, we have omitted the leave-one-out cross-validation for this model. However the visualization obtained when applying this model is depicted in Supplementary Figure 2.

References

[1] Roger A Horn, Charles R Johnson Matrix analysis, 2nd ed. ed. Cambridge University Press, New York, 2013.

[2] Jerome Friedman, Trevor Hastie, and Robert Tibshirani. The elements of statistical learning. Vol. 1. New York: Springer series in statistics, 2001.

[3] Alkes L Price, Nick J Patterson, Robert M Plenge, Michael E Weinblatt, Nancy A Shadick, and David Reich. Principal components analysis corrects for stratification in genome-wide association studies. *Nature genetics*, 38(8):904–909, 2006.

[4] Jian Yang, S Hong Lee, Michael E Goddard, and Peter M Visscher. GCTA: a tool for genome-wide complex trait analysis. *The American Journal of Human Genetics*, 88(1):76–82, 2011.

[5] Michael J Greenacre. Theory and applications of correspondence analysis. 1984

[6] Francis R Bach and Michael I Jordan A probabilistic interpretation of canonical correlation analysis. 2005

[7] Charlotte Soneson, Henrik Lilljebjörn, Thoas Fioretos, and Magnus Fontes Integrative analysis of gene expression and copy number alterations using canonical correlation analysis. *BMC Bioinformatics*, 11:191, 2010.
