# Supplementary figures and images for: Expression reflects population structure
Source: PLoS Genet. 2018 Dec 19;14(12):e1007841. doi: 10.1371/journal.pgen.1007841 (PMC6317812; doi:10.1371/journal.pgen.1007841)

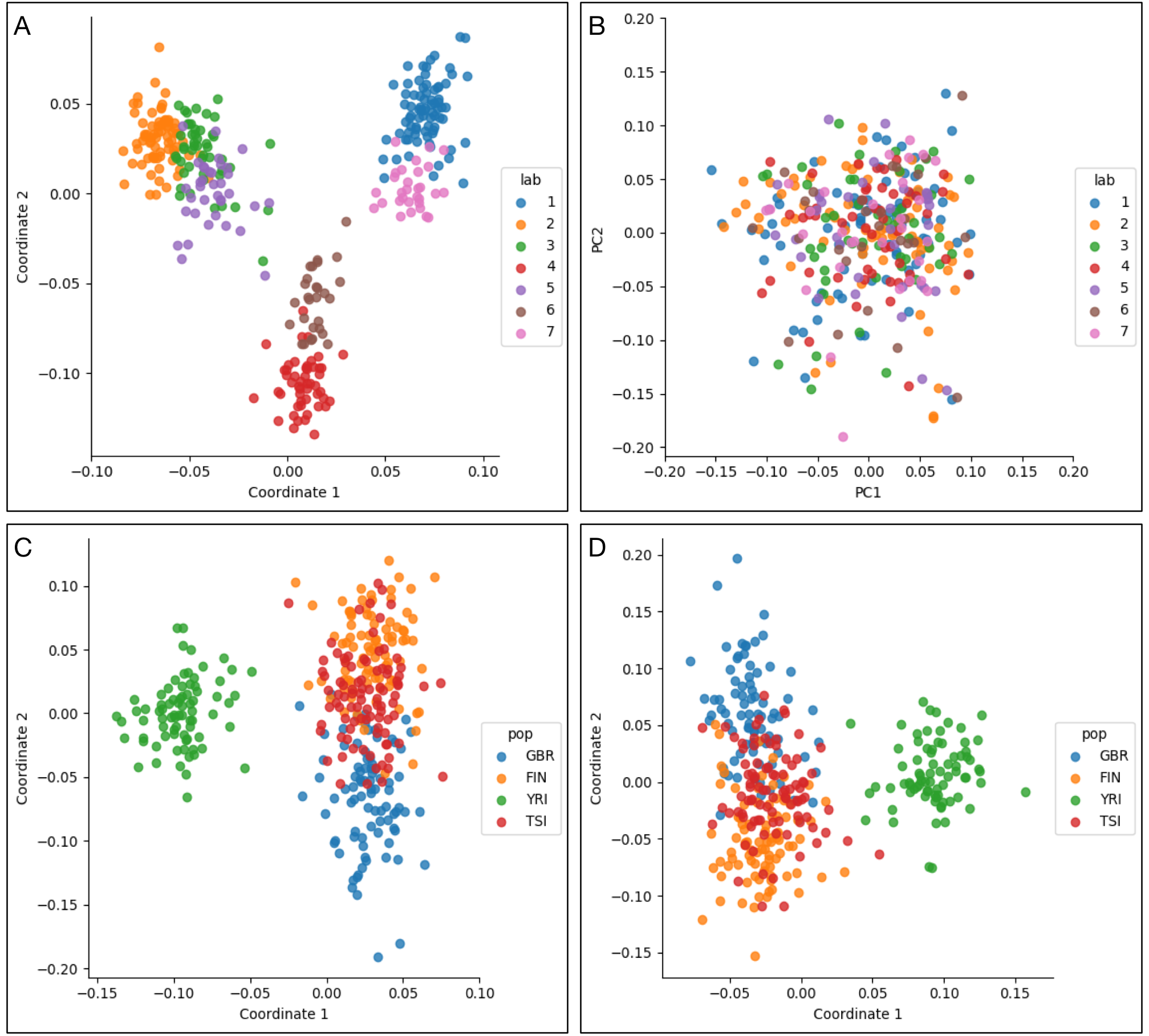

Supplement: S1 Fig — (A) CCA between PCs of expression and a confounding matrix is related to LDA, and projection into the learned space reveals strong clustering by batch within the data. (B) Projecting orthogonally to this space leaves samples scrambled by batch in the first PCs. (C) Using this correction instead of regression gives similar results for CCA between PCs of expression and genotype. (D) As in the main text, structure is maintained during a cross-validation experiment. (PNG) [file pgen.1007841.s002.png]

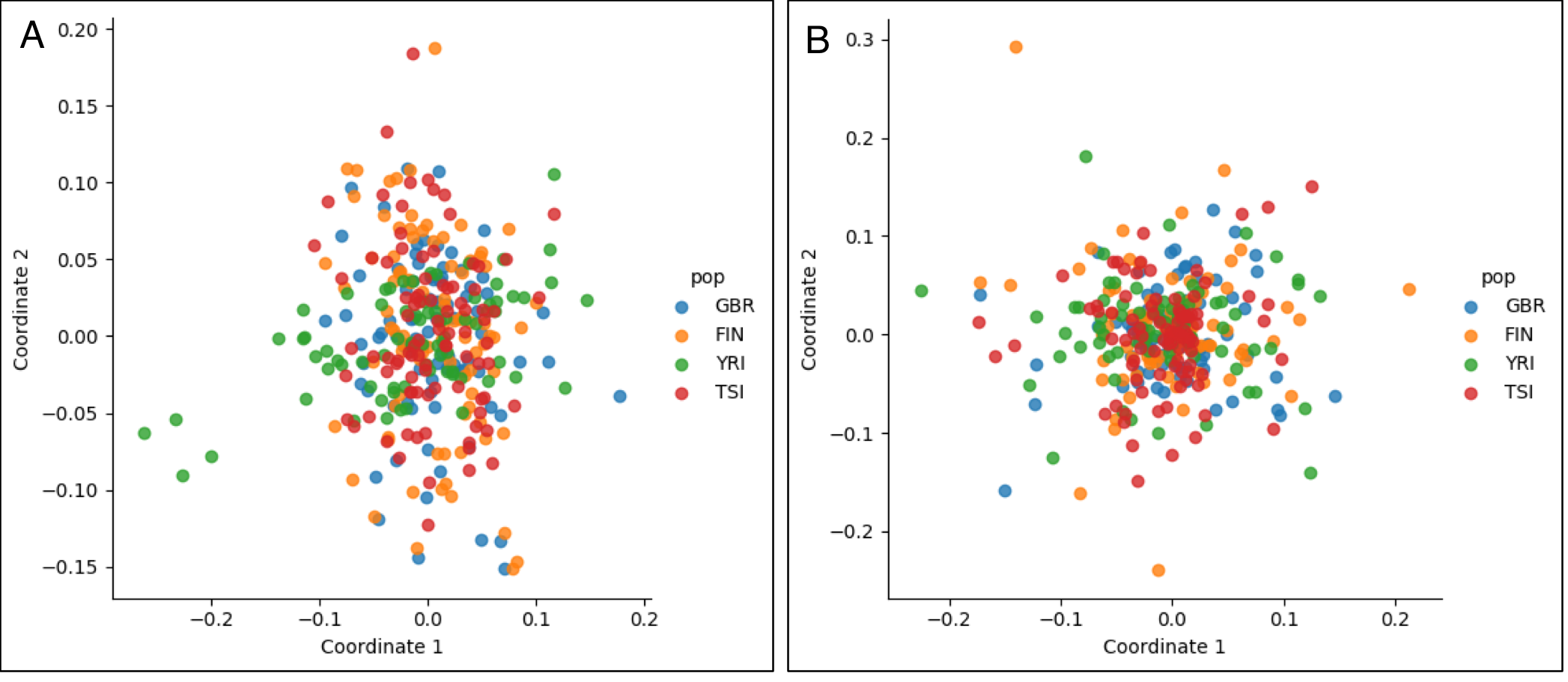

Supplement: S2 Fig — (A) The results of running standard CCA using all genes and genotypes and (B) the CV-projection in a leave-one-out experiment. In this case, no population structure is identified, and the resulting first two correlation coefficients are both 1.0. Since there are many more columns than samples in both datasets, there are many A,B such that Corr(XA,YB) = 1.0. This is an example of extreme over-fitting, with a train error of 0.992 and a test error of 1.295. (PNG) [file pgen.1007841.s003.png]

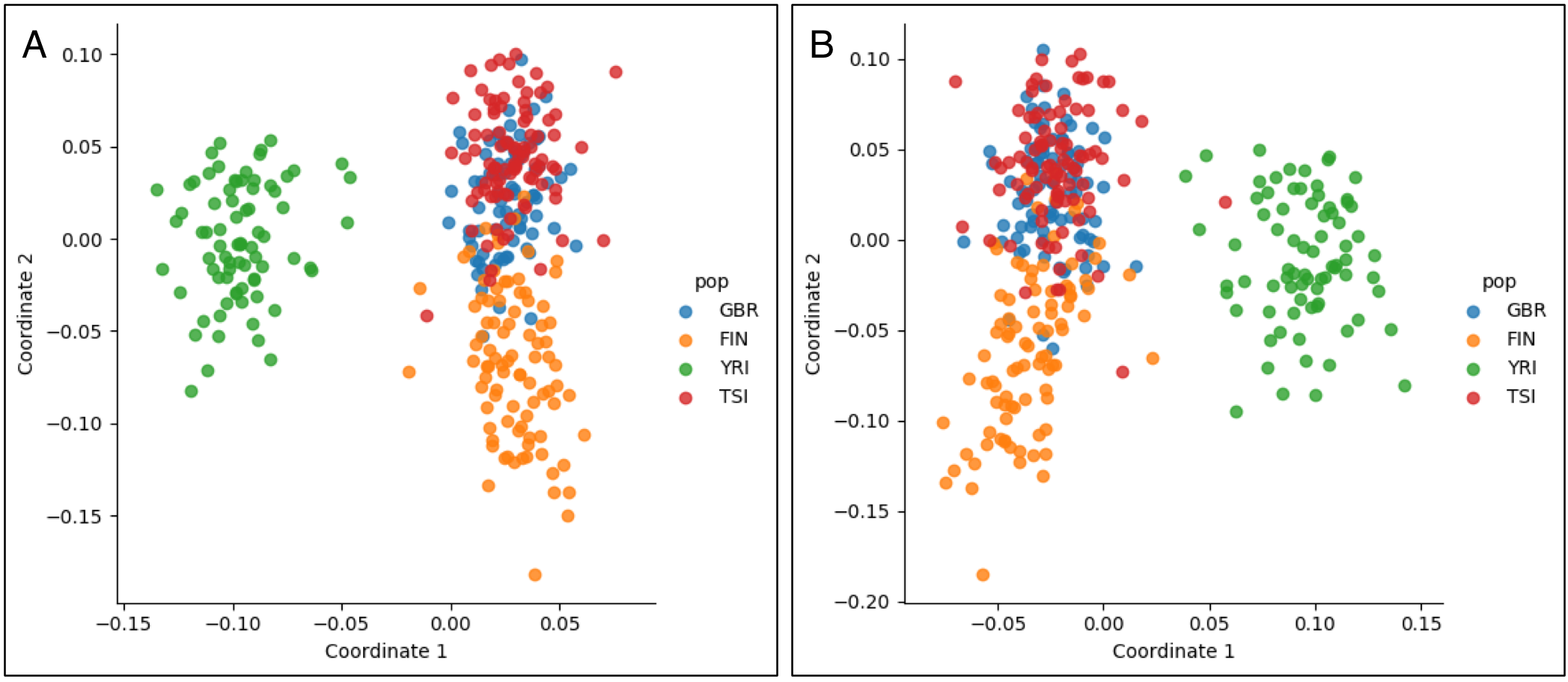

Supplement: S3 Fig — (A) The results of running PCCA without including batch as a covariate. (B) The CV-projection in a leave-one-out experiment on this data. The results are nearly identical to the results when including batch. In this case the first two canonical correlations are 0.964 0.793, respectively. (PNG) [file pgen.1007841.s004.png]

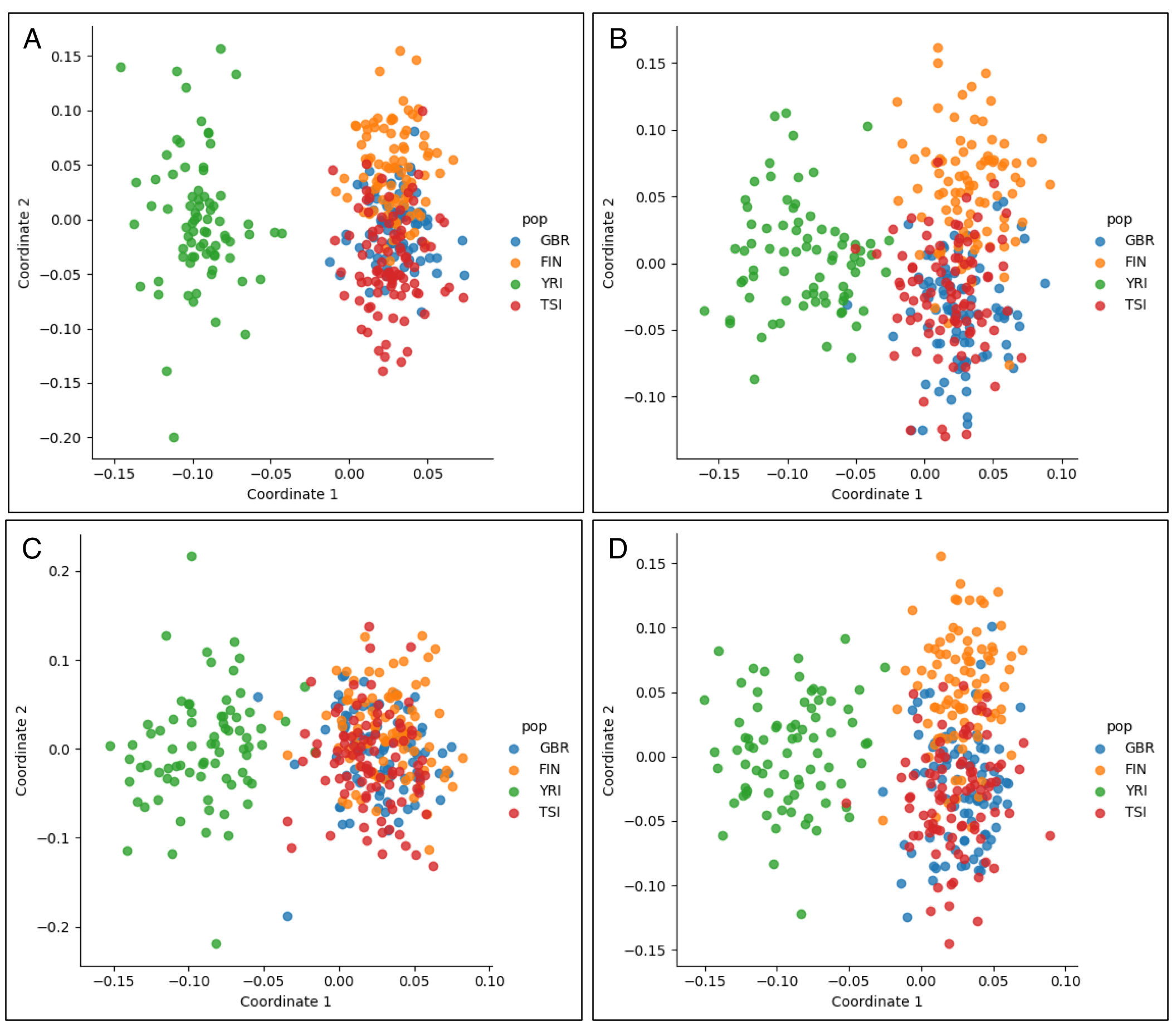

Supplement: S4 Fig — The results of running this procedure when subsampling without replacement either (A) SNPs, (B) genes or (C, D) both. In (A), we sample each SNP with probability p = 0.00001 for a total of 63 SNPs, while keeping all genes, and still observe separation of both the YRI and FIN popultions. In (B), we sample each gene with probability p = 0.01 for a total of 142 genes and again observe similar structure. In (C) we sample each SNP with probability p = 0.00001 and each gene with probability p = 0.01 (57 SNPs, 145 genes) and still observe separation of the YRI, but not the FIN population. In (D), we increase this to p = 0.00002 and p = 0.02, respectively (111 SNPs, 283 genes) and again observe separation of both YRI and FIN populations. (PNG) [file pgen.1007841.s005.png]

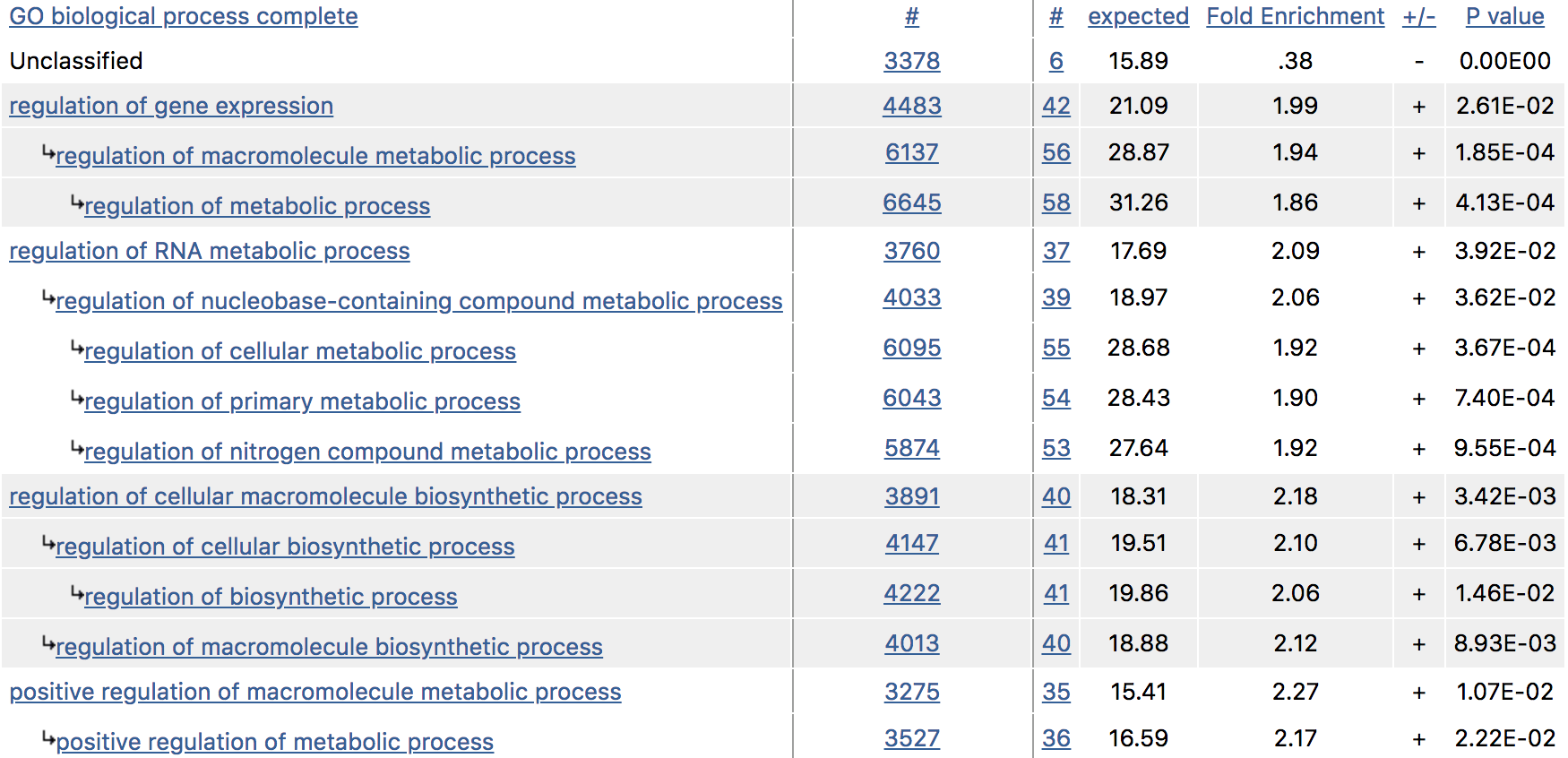

Supplement: S5 Fig — (PNG) [file pgen.1007841.s006.png]

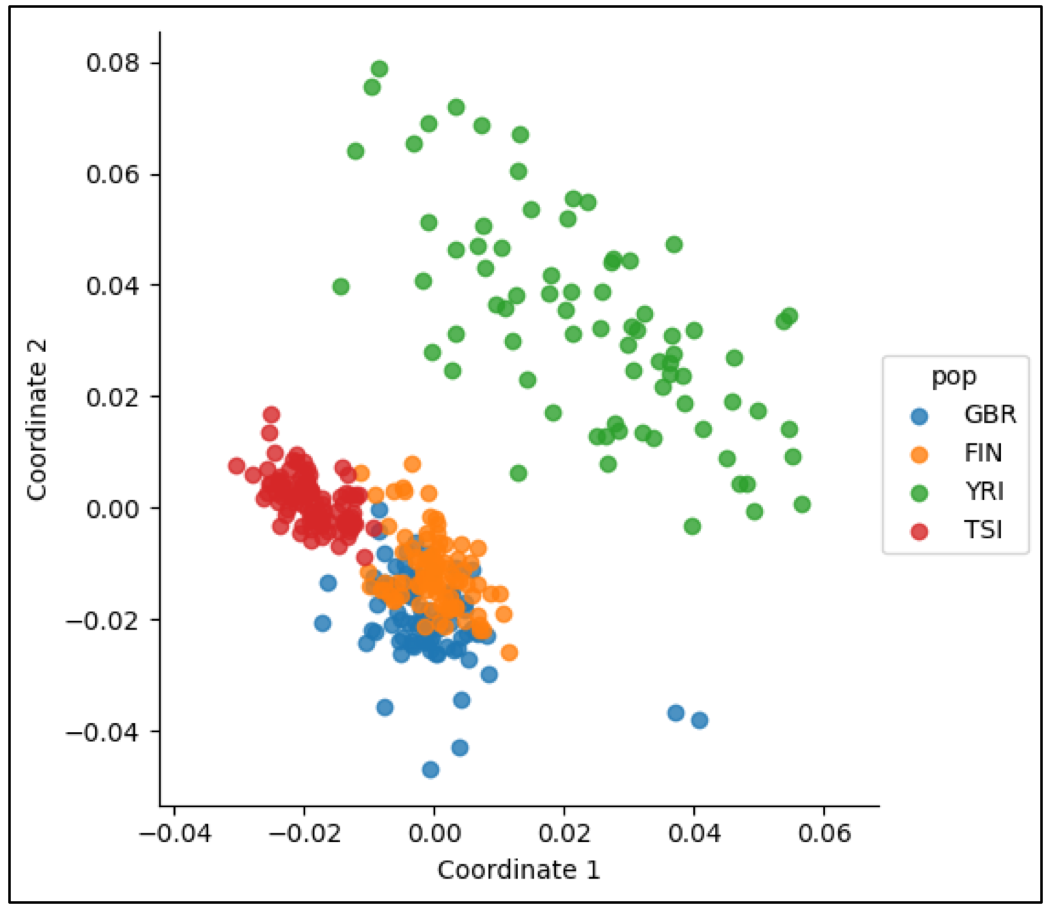

Supplement: S6 Fig — (PNG) [file pgen.1007841.s007.png]

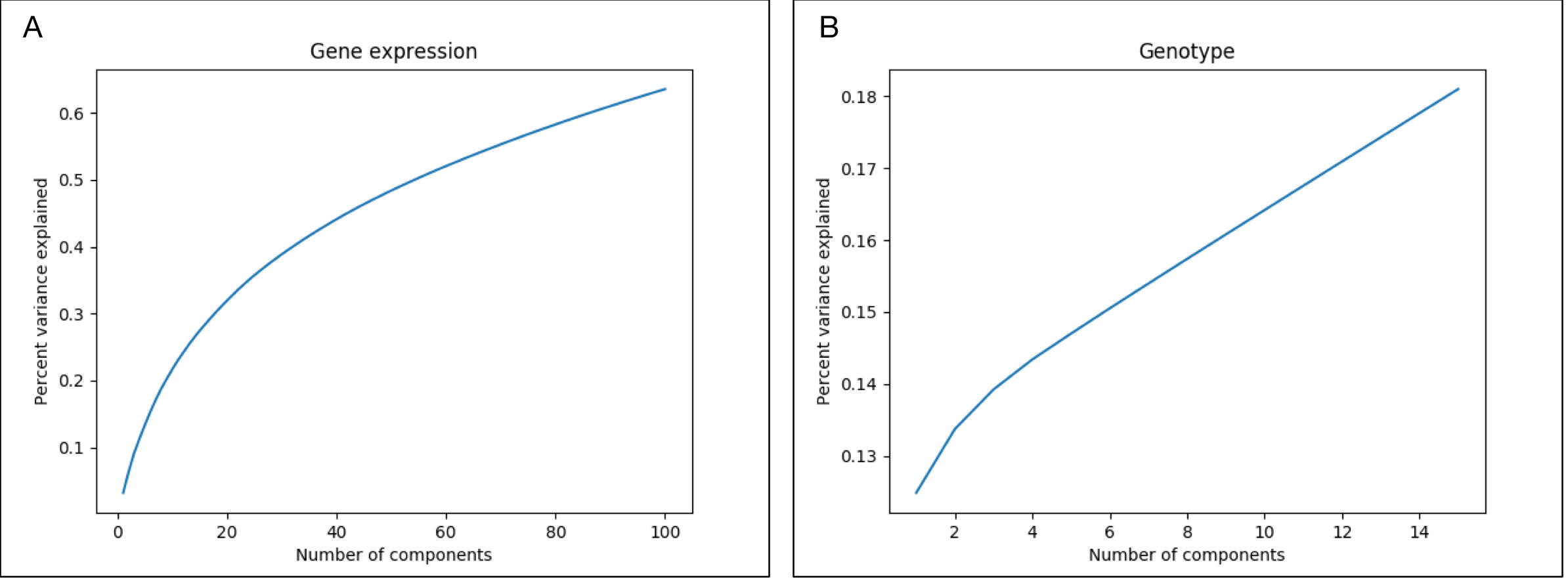

Supplement: S7 Fig — Percentage of variance in the data explained as a function of the number of principal components for (A) gene expression and (B) genotype. The linear region occurs much earlier in the genotype data, implying that fewer components should be used in this analysis. (PNG) [file pgen.1007841.s008.png]

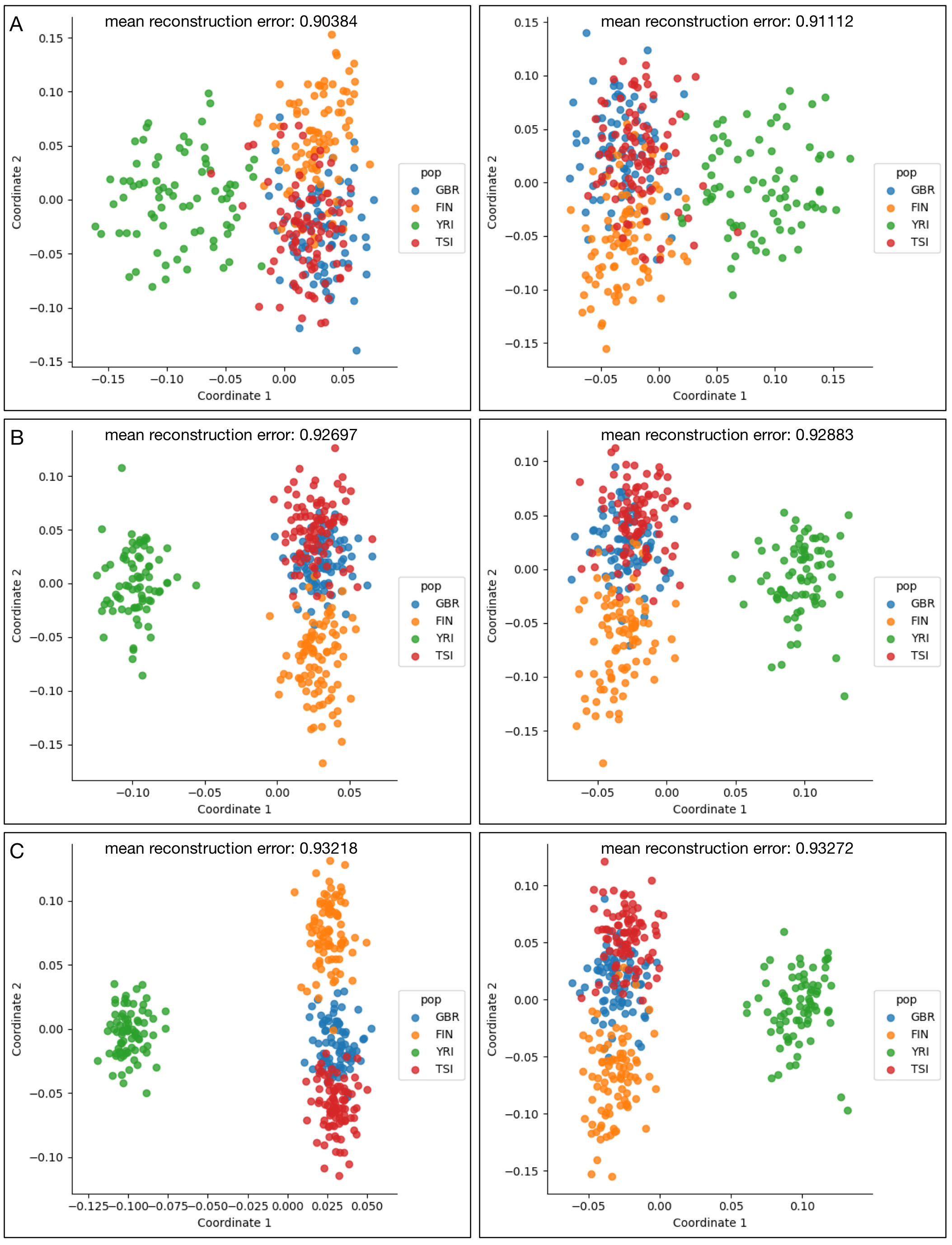

Supplement: S8 Fig — (A) 13 expression and 5 genotype components. (B) 42 gene expression and 15 genotype components. (C) 100 expression and 2 genotype components. (PNG) [file pgen.1007841.s009.png]
